# 2.E. Workshop: The city of proximity: Accessible, Inclusive, Sustainable, Healthy and Salutogenic

**DOI:** 10.1093/eurpub/ckac129.076

**Published:** 2022-10-25

**Authors:** Organised by: EUPHA-URB, EUPHA-ENV, Chair persons: Stefano Capolongo (EUPHA-URB), Marija Jevtic (EUPHA-ENV)

## Abstract

According to the “Urban Health Rome Declaration” at European meeting “G7 Health” that defines the strategic aspects and actions to improve Urban, Environmental and Public Mental Health into the cities, and referring to the Agenda 2030 in which the 11th SDG argue about “Sustainable Cities and Communities. Make cities and human settlements inclusive, safe, resilient and sustainable”, one of the most expressive syntheses of the challenging relationship between urban planning and Public Health is stated by WHO (2016): “Health is the precondition of urban sustainable development and the first priority for urban planners”. Referring to the Healthy Cities & Urban Health definitions, we can consider Public Health not merely an aspect of individual health protection and promotion, but a collective condition, strongly influenced by the environmental context and by the strategies implemented by local Governments. The “Health in All Policies” strategy, clearly underlines how health depend by the quality of outdoor and indoor living environments. In this scenario, healthy living and the requirements for healthy places, infrastructure for the public good and Public Health, cycling, walking, disintegrating the role of polluting traffic from the urban environments, social vulnerability and equality are just a few aspects in complex puzzle when designing the urban spaces for healthy, active, walkable cities. The lockdown due to the pandemic has prevented travels, forcing many people to work at home and reducing the possibility of accessing services in the territory. This condition has further highlighted the importance of urban living areas capable of satisfying basic needs within a reasonably easy range of accessibility. The concept of the “15 minutes city” is a useful vision to represent the city of proximity, where it is possible to meet the needs for sustainable, fair, quality, and healthy living. This dimension of proximity can be central to formulating strategies to improve the quality of urban life. A place of proximity, therefore not only defined based on the physical characteristics and people's uses, but also based on the data collected from a public health perspective in which it is also possible to try to test different types of information and build the conditions to suggest suitable policies and projects. Aim of the Workshop - organized by the two EUPHA Section URB+ENV - it would like to be to build the capacity and knowledge between participants about the main topics and urban features capable to have relevant Urban Public and Environmental Health outcomes. Additional scope is to collected case studies and research experiences considered virtuous at the international level, analyzed in detail to highlight the main urban and architectural features of those healthy experiences and the related health outcomes, such as sedentary lifestyle reduction, increase of the attractiveness of places, reduction of air and noise pollution.

**Key messages:**

• Cities for people, promoting Urban Public Health, Environmental Health and active mobility, require optimization of public spaces for citizens and their activities.

• Case studies and research experiences to highlight the main urban and architectural features of those healthy experiences and the related health outcomes, such as sedentary lifestyle reduction.

